# FAMILY INTERACTION, PSYCHOLOGICAL NEEDS, AND SELF-CARE IN KOREAN MIDAGE TO OLDER CANCER SURVIVORS

**DOI:** 10.1093/geroni/igae098.2728

**Published:** 2024-12-31

**Authors:** Haerim Lee, Sun-ui Shin, Eunyoung Park, Mi Sook Jung, Hyun-E Yeom

**Affiliations:** Chungnam National University, Daejeon, Republic of Korea; Chungnam National University, Daejeon, Republic of Korea; Chungnam National University, Daejeon, Republic of Korea; Chungnam National University, Daejeon, Republic of Korea; Chungnam National University, Daejeon, Republic of Korea

## Abstract

With medical advancements extending the lives of cancer survivors, active self-care becomes increasingly significant. Family interaction and psychological status are keys related to the engagement in self-care of individuals with long-term health issues. This study investigated how family interaction affects psychological needs—namely autonomy, competence, and relatedness—and self-care activities among Korean middle-aged to older cancer survivors. We also examined the variations with age in the roles of family dynamics on psychological needs and self-care behaviors. It is a cross-sectional study utilizing secondary data from two studies, encompassing 277 community-dwelling survivors. We performed descriptive statistics, Pearson’s correlation, and PROCESS macro analysis using SPSS to discern the influence of family interactions. Our results revealed that family interaction plays a crucial role in both satisfying and frustrating psychological needs, which, in turn, directly and indirectly influences self-care activities. Notably, the impact of family dynamics on self-care and the frustration experienced in meeting psychological needs, particularly autonomy and relatedness, was more pronounced in middle-aged survivors than in those over 60. The comprehensive findings highlight the need for healthcare interventions tailored to the age-specific needs of cancer survivors, emphasizing the importance of family engagement in supporting psychological well-being and self-care. The study advocates for strategies to foster active family involvement to enhance overall quality of life and encourage self-care among middle-aged to older survivors.

